# Diagnosis of Peritoneal Carcinomatosis of Colorectal Origin Based on an Innovative Fuzzy Logic Approach

**DOI:** 10.3390/diagnostics12051285

**Published:** 2022-05-21

**Authors:** Valentin Bejan, Marius Pîslaru, Viorel Scripcariu

**Affiliations:** 1 Department of Surgery, Faculty of Medicine, “Gr. T. Popa” University of Medicine and Farmacy of Iași, 700115 Iasi, Romania; viorel.scripcariu@umfiasi.ro; 2 Department of Engineering and Management, Faculty of Industrial Design and Business Management, “Gheorghe Asachi” Technical University of Iași, 700050 Iasi, Romania; mpislaru@tuiasi.ro

**Keywords:** peritoneal carcinomatosis, fuzzy logic, colorectal cancer, carcinomatosis diagnostic, system modelling

## Abstract

Colorectal cancer represents one of the most important causes worldwide of cancer related morbidity and mortality. One of the complications which can occur during cancer progression, is peritoneal carcinomatosis. In the majority of cases, it is diagnosed in late stages due to the lack of diagnostic tools capable of revealing the early-stage peritoneal burden. Therefore, still associates with poor prognosis and quality of life, despite recent therapeutic advances. The aim of the study was to develop a fuzzy logic approach to assess the probability of peritoneal carcinomatosis presence using routine blood test parameters as input data. The patient data was acquired retrospective from patients diagnosed between 2010–2021. The developed model focuses on the specific quantitative alteration of these parameters in the presence of peritoneal carcinomatosis, which is an innovative approach as regards the literature in the field and validates the feasibility of using a fuzzy logic approach in the noninvasive diagnosis of peritoneal carcinomatosis.

## 1. Introduction

Worldwide, colorectal cancer (CRC) represents the third most commonly diagnosed cancer and the fourth most common cause of cancer related mortality [1]. Aside from dissemination through the lymphatic and hematogenous routes, CRC can spread to the peritoneal cavity through transmural invasion and exfoliation of free cells, thus determining peritoneal carcinomatosis (PC) [2]. In the past, PC was viewed as part of the metastatic disease, even in the cases with minimal peritoneal burden, and thus palliative treatment was the only choice. The newer approach considers PC a loco-regional extension of the primary tumor, thus enabling more aggressive surgical and oncological therapy [3]. From the surgical standpoint, extensive cytoreduction surgery through which all macroscopical neoplastic sites are removed, accompanied by intraperitoneal chemotherapy, has greatly improved the prognosis of this once-considered terminal stage [4,5]. However effective, this surgical method is not yet widespread. It requires tertiary or quaternary care hospitals, with specialized surgical staff, and multidisciplinary teams. Another very important requirement is represented by the low peritoneal burden of the patients [6].

Postoperative mortality and morbidity are also relatively high, thus questioning the importance of intraperitoneal chemotherapy compared to solely cytoreductive surgery [7]. Despite advances in oncological and surgical treatment, PC remains associated with poor prognosis and low quality of life [8]. Patients treated with systemic chemotherapy regimens have a mean survival of five to seven months [9,10,11]. The most encouraging reported mean survival was 12.6 months, although caution is warranted. These patients were part of a randomized study and were eligible for cytoreductive surgery and hyperthermic intraperitoneal chemotherapy, thus younger, with low peritoneal burden, and without metastasis [12].

Another issue with modern PC treatment options is related to the limited facilities which can perform this type of surgery and the duration of the diagnostic-cancer care pathway. This duration is of course highly dependent on patient compliance and the effectiveness and organization of the healthcare system. An optimistic duration from the first referral to the general practitioner to the start of therapy is 82 days, although, for 10–25% of cancer patients, this duration can be disproportionately long [13].

The incidence of early PC remains unknown due to low sensitivity imagining techniques and heterogeneity of published data. It is estimated that between 4.3 and 8% of CRC patients present with synchronous PC at the moment of diagnosis [14,15]. Considering the lack of imagistic methods or specific serum markers capable of diagnosing low burden PC, computational intelligence techniques could represent an important aid to clinicians.

Machine learning software integrated with patient databases could be able to identify patients which are likely to present early PC in the case of CRC suspicion. By using certain parameters from the standard blood tests, these systems could be implemented even in primary care, reducing time to referral to specialized centers and thus unburdening secondary and possibly tertiary care centers, and improving time to diagnosis and treatment, improving associated healthcare costs. With quicker referral to specialized centers, the probability of an intermediate intraoperative diagnosis is decreased alongside the improvement of mortality and morbidity.

The aim of the present study is to test if fuzzy logic applied to a patient’s database could diagnose early using non-specific blood test parameters.

Medical diagnosis is a difficult and complex process for the physician who interprets observable signs and symptoms, alongside complementary medical data, various factors, and circumstances. Despite being of greatest importance in treatment and outcome, the duality between subjective and objective is clearly seen [16]. Generally, in order to minimize vague or uncertain factors, which could influence the diagnosis process, medical science has tried to gather empirical data which could later support certain decisional rules. The disease can manifest itself differently in different individuals. One symptom is rarely associated with only one disease. This is true without considering interactions between diseases in the same patient. By using Boolean or binary sets, which are absolute, in the diagnosis process, different uncertainties might be eliminated but also cannot be applied to the human organism, which is by default complex and heterogeneous.

Fuzzy logic on the other hand eliminates the absolute statements from the Aristotelian logic and introduces partial truth values. By doing this, it mimics the human decisional process [17].

Fuzzy logic has proven as a valuable tool in clinical medicine: drug effectiveness [18,19], image processing [20], diagnosis of chronic disease [21] decision making (i.e., estimating treatment response, determining optimal tendon repair technique, or optimizing the choice of radiotherapy course) [22,23,24].

Regarding cancer, fuzzy sets have been used both in detection or diagnosis, and prediction or prognosis [25,26].

Cancers in which fuzzy diagnostic tools have been developed are breast, prostate, lung, renal, and brain cancer [27,28,29,30]. Most of these have used medical imaging as input data, although symptoms or biological findings have also been utilized [29,31].

Regarding colorectal cancer diagnosis, machine learning has been successfully used in different steps and approaches to the diagnosis process. Thus far, the most reliable results come from the use of neural networks, trained with large data comprising of medical images. These neural networks, through pattern recognition, can produce outstanding results with computer tomograph or magnetic resonance images, even in those with less-than-optimal resolution [32,33]. Another novel application is the use of neural networks with colonoscopy or endocytoscopy in correctly identifying colorectal polyps [34,35]. Future applications of fuzzy logic in the pathological diagnosis of colorectal cancer have been hypothesized, yet not developed [36].

Because fuzzy logic mimics the human reasoning and decisional process, fuzzy logic represents a possible solution to complex problems, especially if these problems cannot be accurately represented in a Boolean fashion (i.e., cannot be considered solely true or false) and inherently consist of different degrees of uncertainty. Also, by comprising a multitude of fuzzy rules, fuzzy logic can be used for a personalized approach to each patient. The advantages listed above also simultaneously represent the disadvantages of this approach: difficult and tedious development of fuzzy rules and membership functions, the need for specific medical expertise, and large data [37].

We have not identified any studies which employ fuzzy logic with blood test parameters in the diagnosis of CRC or PC of CRC origin, therefore making the present study the first approach in this field.

## 2. Materials and Method

A retrospective study was performed using data gathered from the observation charts of patients admitted during 2010–2121 in the 1st and 2nd Surgical Clinics, Sf. Spiridon Hospital in Iași, Romania. The data were anonymized and confidential. The study was conducted in accordance with the Declaration of Helsinki, and the protocol was approved by both the Ethics Committees of the Sf. Spiridon Hospital and of the University of Medicine and Pharmacy “Grigore T. Popa” Iași.

Rigorous inclusion and exclusion criteria were applied. Solely patients which were diagnosed with CRC and PC during their current admittance were included in the study. This implies that patients priorly diagnosed with PC or referred to as having a high probability of having PC were excluded. In accordance with the aims of the study, all patients included in the study were diagnosed with PC intraoperatively and supported by the pathology report. Inconclusive pathology results, despite clinical data and intraoperative findings, were excluded from the study. Also, due to its rarity and particular pathophysiology, patients with appendicular mucinous carcinoma were also excluded from the study. Due to the nonspecific nature of the parameters chosen for the study, patients with cancer-related complications (peritumoral abscess, perforation, intestinal obstruction) or other underlying conditions such as concomitant infections were excluded from the study. All patients presented a low peritoneal burden.

Inherently, the strict selection criteria and the subsequent data base characteristics (all patients having a positive diagnosis of CRC) address an interesting question, to which no answer has been reported. Does a multifocal cancer progression (such as PC) determine different systemic homeostasis alterations, mirrored by blood test parameter modifications, as opposed to a single-site cancer development?

Out of the initial data pool, only 74 patients with CRC and PC met the criteria listed above. Similarly, data from patients diagnosed only with CRC was gathered, resulting in a witness group.

The data collected was comprised of 15 parameters. Out of these, only 5 were used in the study: *Age, Hemoglobin (Hb) platelet count (PLT), C-reactive protein (CRP), and alkaline phosphatase (ALP).* The main reason for the choice of these parameters as input data was that they all are a part of a routine blood test. This makes them inexpensive and easily obtainable, especially in the early stages of the diagnosis process, thus in accordance with the aims of the study.

These differences in median value helped define the function intervals. Although appearing to have a clearly different median value, being biological parameters, they are highly heterogenous within and between data sets and the two groups. (Table 1 and Table 2).

Two tail *t*-test using unequal variances were performed on matching parameters of both sets to evaluate statistical significance. We considered a 95% confidence level (Table 3).

We considered the null hypothesis as the absence of statistical significance or relationship between the two datasets. As seen in Table 3, for three parameters the null hypothesis has been rejected. In the case of Hb, the evidence against the null hypothesis is weak, thus being considered a trend. The null hypothesis can be considered valid in the case of age, meaning there is no statistical significance between the two sets. Keeping this in mind, all the parameters above have been used in the modeling because of their medical significance. Each of the mirrors a different interaction between cancer and host, thus useful in a complex multi-sided approach.

## 3. Results

### 3.1. Fuzzy Modelling

**Figure 1:**
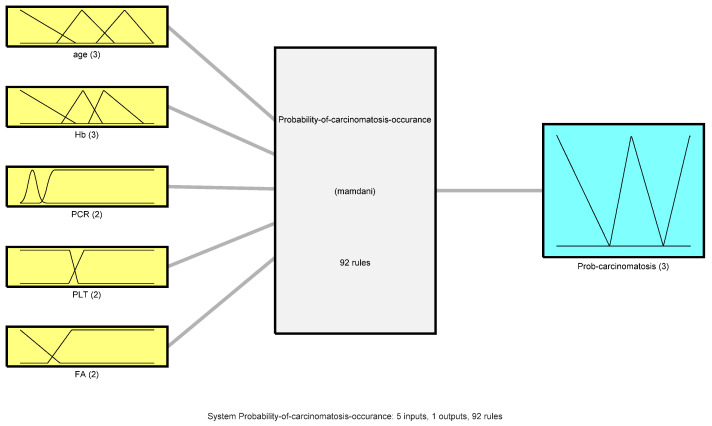
Fuzzy system for probability of carcinomatosis occurrence.

The fuzzy model takes into consideration age alongside four of the relevant biological parameters associated with the presence of cancer: *age*, *Hemoglobin level*, *C-reactive protein CRP, platelet count (PLT)*, and *alkaline phosphatase (ALP)*. The reasoning behind this particular choice of parameters was that each one mirrors the changes occurring through different systems and subsystems. For the inference, it was used the MATLAB Fuzzy Logic Toolbox because it generally provides complete environments for building and testing fuzzy systems and very friendly graphical user interfaces. Consequently five-input one-output problem is to be discussed and solved in order to determine the probability of carcinomatosis occurrence (Figure 1).

The fuzzy modeling algorithm implies the following steps:

- Step 1: Fuzzification

**Figure 2:**
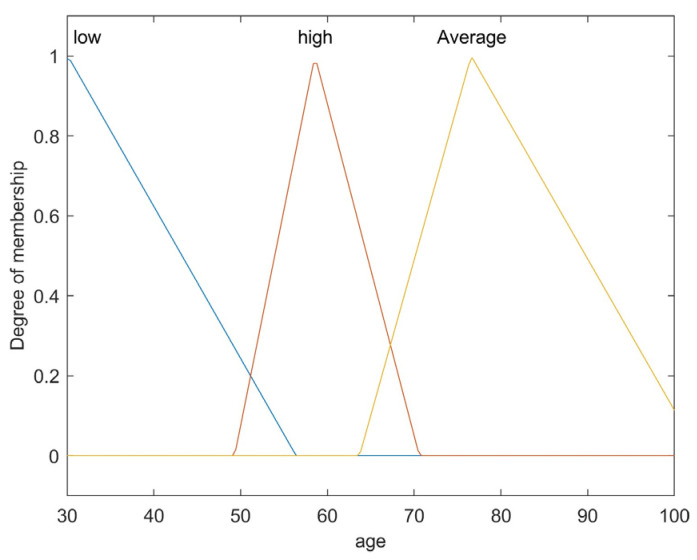
Membership function of age as input variable.

**Figure 3:**
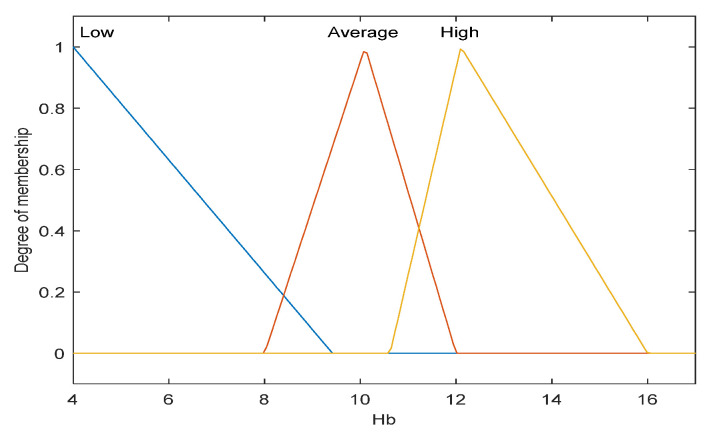
Membership function of *Hb* as input variable.

**Figure 4:**
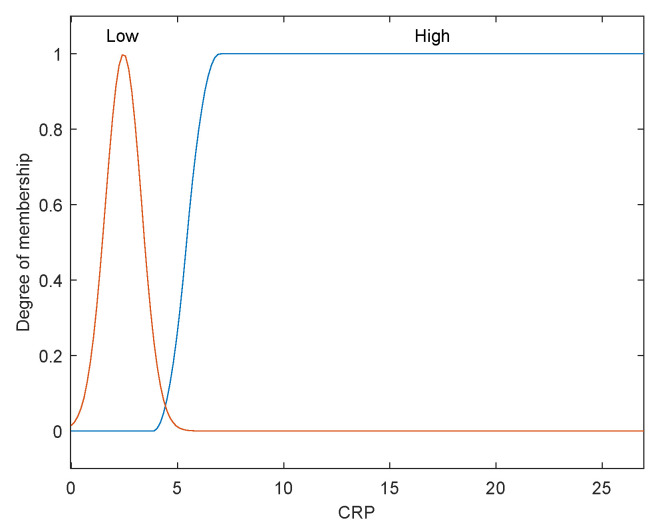
Membership function of *CRP* as input variable.

**Figure 5:**
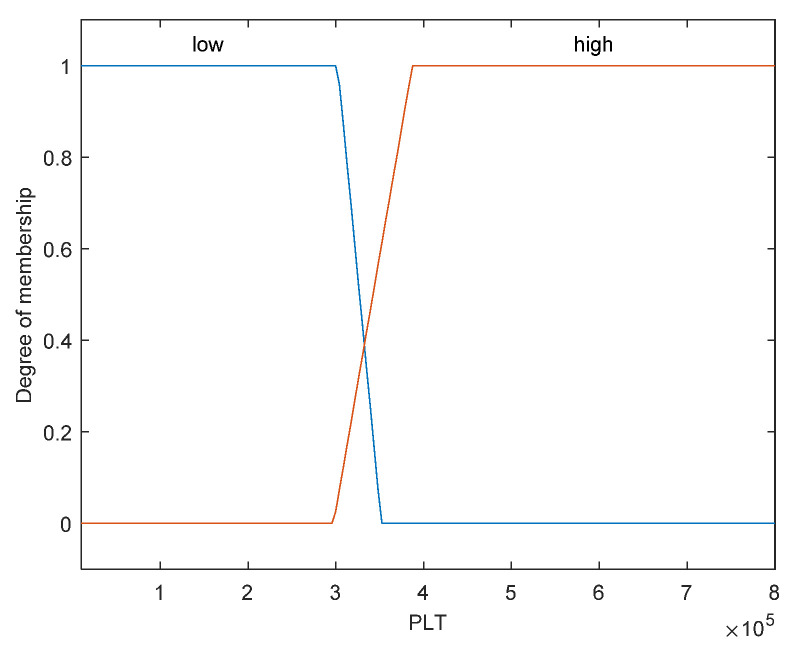
Membership function of *PLT* as input variable.

**Figure 6:**
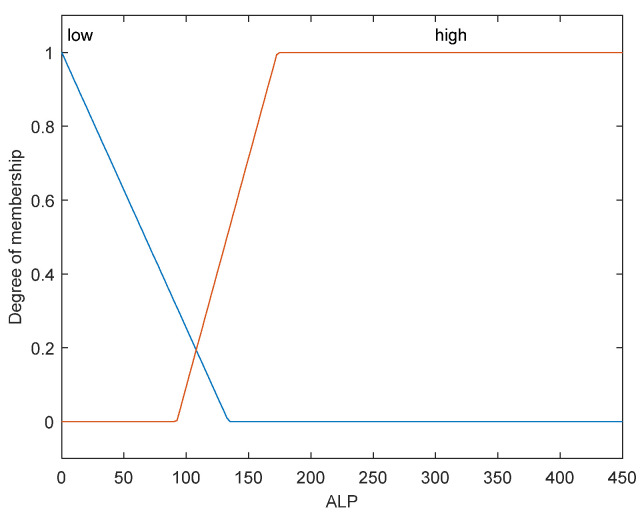
Membership function of *ALP* as input variable.

The first step is to take crisp inputs, *age*, *Hb*, *CRP*, *PLT*, and *ALP*, and to determine the degree to which these inputs belong to each of the appropriate fuzzy sets. The crisp input is always a numerical value limited to the universe of discourse. The ranges of the universe of discourses were determined by normal range values. Fuzzy sets can have a variety of shapes. However, for our modeling, we have chosen a triangle, trapezoidal-shaped, or a Gaussian curve membership function to provide an adequate representation of the medical expert knowledge, and existing medical data and, at the same time, significantly simplifies the process of computation. In Figure 2, Figure 3, Figure 4, Figure 5 and Figure 6, the shapes of membership functions used as the linguistic variables in the fuzzy model are presented. For all the input parameters, in order to respect scientific realities, and at the same time for a smoother response to modeled fuzzy system, an adequate overlap in adjacent fuzzy sets was maintained.

There are used three triangular linguistic levels for *age* and *Hb: Low, Average*, and *High* and two trapezoidal and Gaussian linguistic levels for *CRP, PLT*, and *ALP* as input variables. For the output variable *prob-carcinomatosis*, we used three linguistic levels *Low*, *Average*, and *High*. These linguistic levels were chosen based on existing medical data, both from literature and from our data sets, medical specialist experience, and because they are adequate for Mamdani fuzzy inference system. The unique knowledge of the process is captured by membership functions for each variable through the use of these specific linguistic levels. For example, the *age* behavior is represented by the three triangular functions shown in Figure 2, where the *y*-axis represents the membership function value between 0 and 1 and the *x*-axis represents the patients’ age range between 30 and 100 years, which is the full interval of age values, as found in our data sets and partially projected with the help of described incidence patterns. In Figure 3, Figure 4, Figure 5 and Figure 6 the parameters were represented based on the data that were collected both from medical sources and the present patient data.

**Figure 7:**
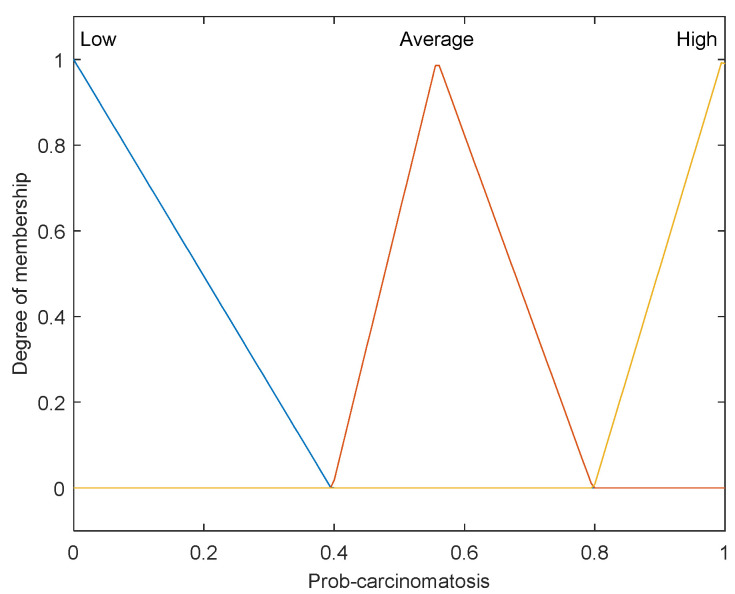
Membership function of *Prob-carcinomatosis* as output variable.

The three overlapping membership functions for *age* have been defined in an explicit manner using the three linguistic variables: *low, average*, and *high*. Therefore, the *low age* linguistic variable represents the values interval under 57 years old. The broadest values interval is associated with the *average age* linguistic variable, ranging between 65 and slightly above 100 years old, with the strongest influence of age around 75 years old, afterwards following a descending slope, simulating the incidence pattern. The highest risk interval is defined by the linguistic variable *high* and is represented by the values range between 50 and 70 years old, with the highest level of membership around 60 years old. The membership function for the *Hb*, as shown in Figure 3, has been modeled in a similar fashion using also triangular linguistic levels, but with a slightly different shape of the membership functions for low, average, and high. In the case of the output variable—probability of carcinomatosis (*Prob-carcinomatosis*)—the low, average, and high triangular membership functions were also preferred for the visual representation, but in this case, no overlapping between the functions has been modeled. This decision was taken in order to simplify the interpretation of the output data sets by a medical professional unaccustomed to *soft computing* (Figure 7).

- Step 2: Rule evaluation

The next step in fuzzy modeling consists in applying already fuzzified inputs to the antecedents of the fuzzy rules.

The very fundamentals of fuzzy logic and approximate reasoning are conditional statements based on fuzzy rules. The simplest fuzzy rule can be presented in the following form: **if** A **then** B. However basic it might appear, this denotes an involvement operation from *antecedent* or *premise* A to *consequent* B [38].

The writing down of rules is based on years of medical experience and data from medical literature. Obviously, the number and shape of membership functions are subject to medical characteristics discussed in the paper. To obtain fuzzy rules the medical expert was asked to describe the probability of carcinomatosis occurrence using the fuzzy linguistic variables defined previously.

An example of rules for the modeled example is given below:

*1*  *If (age is low) and (Hb is Low) and (PCR is Low) then (Prob-carcinomatosis is Low) (1)*
*2*  *If (age is low) and (Hb is Low) and (PCR is High) then (Prob-carcinomatosis is Average) (0.5)*

*91*  *If (age is high) and (Hb is High) and (PCR is High) and (PLT is high) and (FA is low) then (Prob-carcinomatosis is High) (1)*
*92*  *If (age is high) and (Hb is High) and (PCR is High) and (PLT is high) and (FA is high) then (Prob-carcinomatosis is High) (1)*

At the end of each rule, a weight is attached, representing the importance that is given to each rule. The range of the weight varies in range [0, 1], value 1 signifying the highest importance. As can be observed, for some of these rules the weight is equal to 1 and for others is equal to 0.5 (noted in parenthesis) according to medical experts and scientific literature.

In order to analyze the data in our model, it is necessary to investigate the logical connectives, namely negation (NOT), conjunction (AND), and disjunction (OR) used in Fuzzy logic [39]. For our study, the connective used is represented by AND operator to connect the propositions and because it better serves the intended purpose.

In case the antecedent of a rule involves the use of multiple parts then the overall degree of truth must be triggered. Let us take, for instance, the rule 2 as follows:

*If (age is low) and (Hb is Low) and (PCR is High) then (Prob-carcinomatosis is Average)* (0.5)

The rule has three antecedents ((*age is low*) and (*Hb is Low*), and (*PCR is High*)) which must be evaluated and computed, resulting in a single number. Consequently, the degree of truth for the antecedent is based on the fuzzy operator AND application of the three parts of the antecedent.

In case of a more complicated antecedent construction is required, the precedence of fuzzy operators should be defined or grouping operators should be used.

- Step 3: Aggregation of the rules

At this stage, the fuzzy output sets derived from fuzzy rules in the fuzzy inference system must be computed to create an output, namely the probability of carcinomatosis occurrence. The process of combining all these output fuzzy sets is called *aggregation*.

The literature specifies many methods of aggregation but the most commonly used are *maximum, probabilistic, and/or sum (bounded sum).*

**Figure 8:**
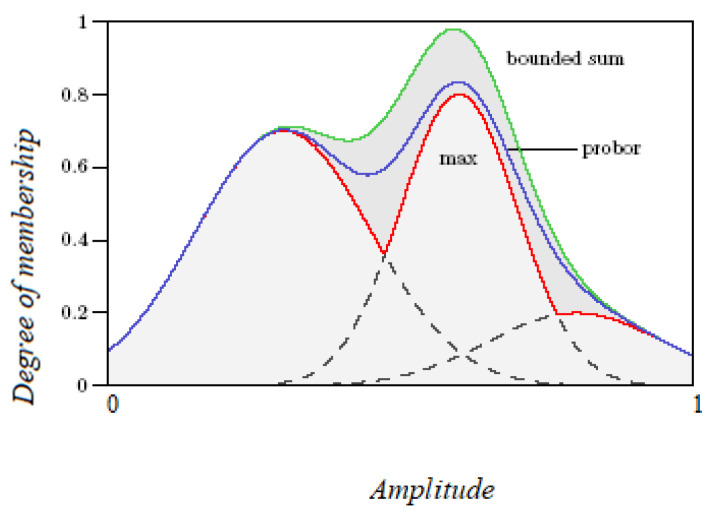
Three methods of fuzzy set aggregation.

These three methods are shown in Figure 8. Choosing the most appropriate method depends on the model requirements and application type but, nevertheless, the fuzzy mathematics used must be consistent [40].

Taking into account the rule 80 formulated for the actual approach, Figure 8 shows the application of the fuzzy operator AND aggregation of the rule for *Prob-carcinomatosis.*

Although *probabilistic* and *sum* methods provided slightly different results for our modeling and based on medical expert opinions the most suitable method was *maximum* aggregation. Because in a fuzzy system, every rule triggers to some degree, the antecedent is true to some degree of membership, leading thus the consequent is also true to that same degree [41].

**Figure 9:**
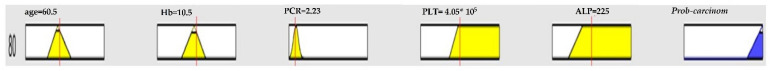
Application of AND operator for rule aggregation.

Figure 9 depicts the application of AND operator in order to compute the consequent (*prob-carcinom*). The output value of a consequent’s degree of truthfulness can be estimated directly from a corresponding degree of truthfulness of the antecedent.

As mentioned, the fuzzy rule number 80 has multiple antecedents (or multiple parts of antecedent), and based on fuzzy operator (AND), a computation is performed to obtain a single number representing the result of the antecedent evaluation [42].

Once the number computed (the truth-value) is applied to the consequent membership function, in this case, the membership of *prob-carcinom*.

- Step 4. Defuzzification

Defuzzification represents the final step of the fuzzy inference process. By fuzzification, the rules are assessed but for our case, a crisp number representing carcinoma probability occurrence must be obtained. In terms of input, regarding this last stage, we can consider the fuzzy aggregate output set, and the output following defuzzification is a single number.

**Figure 10:**
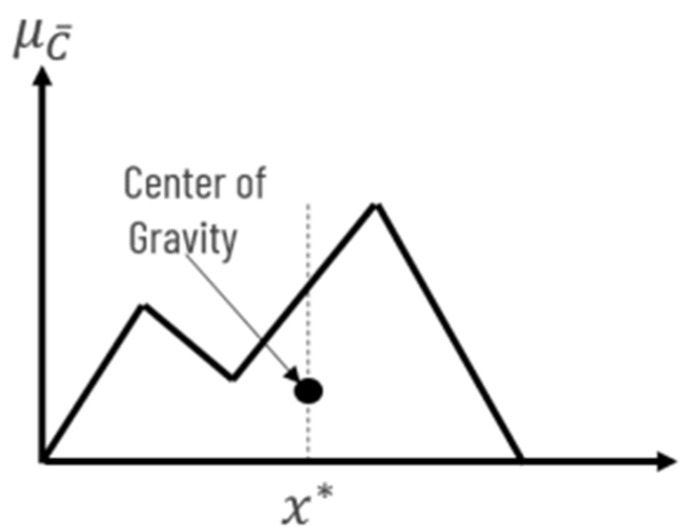
Defuzzification using CG method.

The literature presents a couple of defuzzification methods (*center of gravity, bisector, middle of maximum, largest of maximum, smallest of maximum, and average*) [43]. Besides the fact that center of gravity seems to be one of the most popular methods nevertheless this method was chosen because it is very well suited to the particularities of our system. Center of Gravity (or centroid technique) (CG) is the most prevalent and physically appealing of all the defuzzification methods [44]. The basic principle of the CG method is to find the point $x^{*}$ where a vertical line would slice the aggregate into two equal masses—Figure 10.

$\mu_{\bar{c}}$ being defined with continuous membership functions mathematically, the center of gravity (CG) can be expressed as in Equation (1):

(1) $$x^{*}=\frac{\int \mu_{\bar{c}}\left(x\right)\cdot xdx}{\int \mu_{\bar{c}}\left(x\right)dx}$$

where $\int \mu_{\bar{c}}\left(x\right)dx$ denotes the area of the region bounded by the curve $\bar{c}$.

A complete example of a fuzzy inference system is now provided. In this example, five input linguistic variables (Age, *Hemoglobin level*, *C-reactive protein CRP*, *platelet count (PLT)*, and *alkaline phosphatase (ALP)*) and one Mamdani outputs (*prob-carcinom*) are used. There are ninety-two rules given in Rule evaluation, while the implication methods are the classical fuzzy ones (max/min) and the centroid method of defuzzification is used.

The fuzzy Logic Toolbox displays a roadmap of the whole fuzzy inference process. The first five columns of plots (the yellow plots) show the membership functions referenced by the antecedent, or the **if**-part of each rule. The sixth column of plots (the blue plots) shows the membership functions referenced by the consequent, or the **then**-part of each rule. The last plot in the sixth column of plots represents the aggregate weighted decision for the given inference system.

This carcinomatosis probability will depend on the input values for the system. The defuzzified output is displayed as a bold vertical line on this plot.

The input parameters and their current values are displayed on top of the columns. The values can be adjusted and traced through the movement of the red index line horizontally, each time a new calculation is performed, and so the whole fuzzy inference process is retaking place. A yellow patch of color under the actual membership function curve is used to make the fuzzy membership value visually apparent.

**Figure 11:**
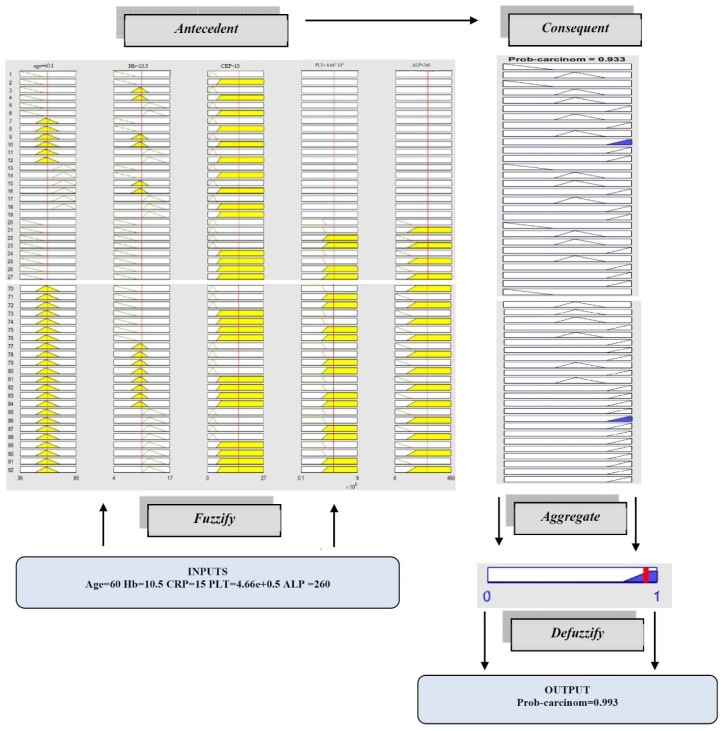
Fuzzy inference system.

Figure 11 shows the overall inference system provided with some particular values assigned to input parameters. The inputs are given for *Age* (60) and *Hemoglobin level (10.5), C-reactive protein (15), platelet count (4.66 × 10^5^), alkaline phosphatase (260)*, and the vertical red lines indicate the input values. The number of rules is 92, but in the figure are presented only the first 12 rules and the last 12 due to obvious reasons, as the rules were computed in MATLAB Logic Toolbox. The route that the data follows in order to be assessed and computed is the following: firstly, the inputs are fuzzified being transformed into fuzzy sets. Once these sets become antecedents, they are processed by applying the logical connective. For every rule, depending also on the weight assigned, the implication is performed, resulting in a scaled output set. The red lines represent a sweep of the input parameters over the entire reference range, the intersection with membership functions (yellow area) presenting at the same time the fuzzification process and generating antecedents. The blue area represents the consequents (result) of each implication and the output fuzzy set. Afterward, as mentioned, in order to get a crisp number, the aggregation and defuzzification processes are applied. So, for our particular example, the final output fuzzy set is defuzzified to produce a value of 0.922 for *Prob-carcinom*, in regard to the above-mentioned input parameters values.

### 3.2. Evaluation and Fuzzy System Tuning

In our model, the last and the most difficult task was tuning the fuzzy system to assess and observe if the system fulfilled the requirements underlined at the beginning. The fuzzy Logic Toolbox can generate a surface in order to help the user to analyze the system’s performance. The probability of carcinomatosis occurrence (*prob-carcinom*) can be described in a 3D figure taking into consideration two of the five input parameters depending on the user’s interest.

**Figure 12:**
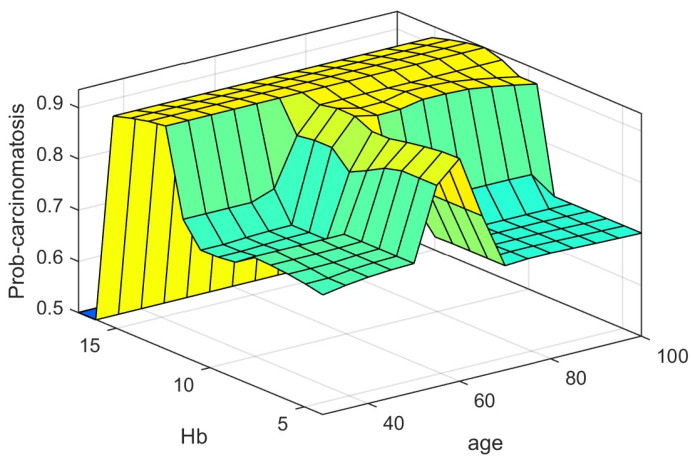
Prob-carcinom variation with Hb and Age.

**Figure 13:**
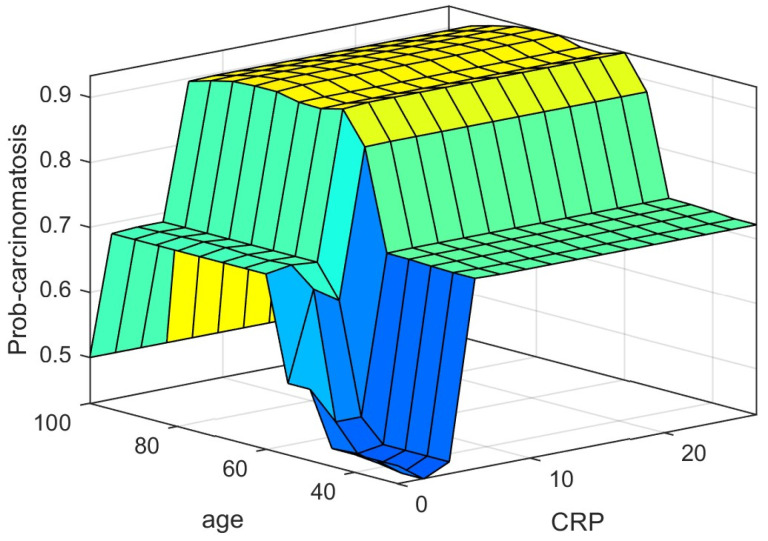
Prob-carcinomatosis variation with Age and CRP.

Figure 12 and Figure 13 represent three-dimensional plots for two input and one output (Prob-carcinom) systems. On the one hand, the distribution of the estimates of *prob-carcinom* based on *Hemoglobin level* and *age* is presented in Figure 12, and on the other hand based on *age* and *C-reactive protein* is presented in Figure 13.

The fuzzy Logic toolbox can generate a three-dimensional output surface by varying any two of the inputs and keeping other inputs constant. Thus, in Figure 12, the performance of the system can be observed: green areas are associated with a low level of carcinomatosis probability occurrence determined by low values of *Hb* and low values of age; opposite, yellow areas are associated with a high level of carcinomatosis probability occurrence determined by high values of *Hb* and average values of *age*. In Figure 13, high levels of carcinomatosis probability occurrence are associated with average values of *age* and high values of *CRP*.

Of course, similar explanations can be provided, as we mentioned before, for every two parameters out of five.

Hence the model takes into consideration all the limits presented before in prior literature and comprises key indicators transformed into commensurable units, with the objective to integrate them into a single measure in order to generate an adequate carcinomatosis probability assessment in regard to the commensurability issue. A performant carcinomatosis probability assessment level requires good results associated with indicators because they cannot hide deficiencies of policies or processes—in regard to fungibility issues.

## 4. Discussions

Machine learning software integrated with patient databases could be able to identify patients which are likely to present early PC in the case of CRC suspicion. Using certain parameters from the standard blood tests makes this method applicable from the moment of the first presentation. The extremely rigorous selection criteria of patients have been applied in order to maintain the scientific accuracy of the medical data. This decision ultimately led to a small database, comprised of medical information of patients with a positive diagnosis of both CRC and PC. A further tuned version of this fuzzy system would be valuable on all levels of healthcare. The clinical suspicion of CRC could be established by the general physician in primary care when confronted with certain situations, e.g., a palpable mass found during the rectal digital examination or patients associating weight loss and lower gastrointestinal symptomatology. Therefore, this type of diagnostic fuzzy system could be implemented even in primary care, reducing time to referral to specialized centers and thus unburdening secondary and possibly tertiary care centers, thus improving time to diagnosis and treatment, and improving associated healthcare costs. With quicker referral to specialized centers, the probability of an intermediate intraoperative diagnosis is decreased alongside the improvement of mortality and morbidity.

- Age

The incidence of CRC follows a steady ascending trend starting with the age of 40, then after the fifth decade of age, an abrupt progressive incidence increase can be observed, with 90% of all cases being diagnosed after the age of 50 [45]. Patients in their 60′s and 70′s are 50 times more likely to be diagnosed with CRC than patients in their 40′s [46]. Newer populational studies have shown an increase of incidence in young patients, already being in the first 10 cancers diagnosed between the ages of 20 to 39 [47].

CRC in young patients, usually defined as adults under 40, presents several particularities. Approximately 66% of cases are diagnosed in the late stages of the disease, as opposed to 32–49.2% of patients over 40 [48]. This can be attributed to a delay in diagnosis due to lower overall incidence, but also to the histological types. In the young patients, there is a predominance of poorly differentiated or signed cell tumors, both associated with high tumoral aggressiveness and thus poor prognosis. These two histological types account for 27% and 21% of all CRC types, as compared to the general population where the incidence of these histological types are 2–29%, respectively 1–15% [49,50].

The five years survival rates are also low: 33.4% (with important variability between 0 and 60% depending on the study methodology) [51,52]. Comparatively, they present a better five-year survival in the early stages of diseases, secondary to better tolerability to chemotherapy and aggressive surgery [53].

The present fuzzy logic system has been modeled using the age interval 30 to 100. This was not an arbitrary decision, and it represents part of the limitations of the study. The system was designed using data available from the medical literature, study database, and medical expertise. Although CRC has an established incidence pattern described by epidemiological studies, the data regarding the incidence of isolated PC is limited, mainly due to difficulties in diagnosis in cases with a low peritoneal burden. We have not found, through enquiry of medical databases, an incidence pattern for patients under 40 years of age with PC of CRC origin. In our own databases, only three cases were under this threshold, of which one case with PC. For these reasons, in accordance with data from epidemiological studies and our patient’s medical database, we have modeled the fuzzy system from a probabilistic standpoint, using CRC incidence patterns, the highest risk being the age interval 50 to 70 years old. Future populational studies might unravel incidence patterns for PC under the age of 40 which could, in turn, be used for further tuning of the system, i.e., using an additional membership function.

- C-reactive protein

More than 150 years ago, the connection between the immune system and cancer was stated for the first time. This was due to the direct observation of leucocytes in neoplastic tissue, by Rudolf Virchow [54]. This interaction became an ongoing research topic, and despite the important volume of information gathered, especially in the last three decades, it remains to be fully comprehended [55]. The role of the immune system is to defend the host against pathogens or transformed cells. The immune system comprises two different interlinked sub-systems: the innate and the acquired immune system. The innate immune system or the nonspecific first line of defense is represented by dendritic cells, macrophages, mast cells, neutrophiles, eosinophiles, basophiles, and natural killer (NK) cells, whereas B and T CD4+ and CD8+ represent the acquired immune system.

The initial theory was that the immune system protects the host by targeting the cancer cells, which, during carcinogenesis, lose cellular traits becoming non-self. This was referred to as immunosurveillance [56]. This hypothesis, although confirmed by numerous experimental and observational data, can only partly describe the interaction. In the three-stage model of immunoediting, the first phase of the elimination phase corresponds to immunosurveillance. If this first phase is incomplete, and thus some cancer cells survive the initial immune assault, the second phase called equilibrium will begin. This second stage is an extremely dynamic phenomenon in which, because of the intense selection pressure, many unstable tumor cell variants will be destroyed, and new variants will arise, capable of resisting the immune attack. The third stage of immunoediting, called escape, is characterized by the inefficiency of the immune system to contain the neoplastic process [57]. The most intriguing paradigm shift is the dual role of the immune system inside the tumoral microenvironment. Depending on context and tumor type, immune cells can either suppress or promote tumorigenesis [58].

C-reactive protein (CRP) represents the prototypical acute phase serum protein. It is synthesized mainly in the liver in response to interleukin-6, and synthesis is enhanced by IL-1 β. The main role of RCP is inflammatory response regulation, either through anti-inflammatory or pro-inflammatory effects. During the acute phase, CRP is produced rapidly and at high levels [59].

Considering the immune system-cancer interaction and the existence of a serum marker that mirrors the systemic response to inflammation, the next logical step would be using this marker in cancer diagnosis or prediction. However, multiple issues arise. The first would be determining a cut-off value, above which the marker gains clear clinical significance [60].

The second issue is regarding the innate immune system which determines a nonspecific response of the host. In order to consider CRP as a diagnosis marker, all other reasons for an inflammatory response must be excluded. Also, using CRP as a prognostic index seems to be more adequate considering the more consistent data associations, as opposed to cancer diagnosis or staging [61].

For our model, we have chosen two membership functions defined by the linguistic variables: *low* and *high*. Only two membership functions have been defined in order to mimic the distribution pattern found in medical practice. This particular parameter has only two values intervals: normal value range and pathological (or above normal) values range. The first membership function, *low*, comprises the normal value range and the values below 5 mg/dL, values which can describe a low intensity immune response, as found in our non-PC database, and confirmed by medical literature data.

- Platelets

One of the first mentions (in western medicine) of abnormal platelet activity in neoplasia was made in a lecture by Armand Trousseau in 1865, describing *Phlegmasia alba dolens*. Through case series, he described migratory deep vein thrombosis as a prognostic factor of occult neoplasia [62]. Since this early mention, multiple studies have proven the association between deep-vein-thrombosis/venous thromboembolism and cancer, high risk being in the first months after diagnosis or in the presence of metastasis [63].

Tumors trigger hemostasis through multiple mechanisms. Tumor cell by-products upon entering the blood stream can initiate intravascular coagulation. This process is amplified if during the invasion phase the extracellular matrix is exposed or if the platelets interact with circulating tumor cells [64]. Apart from the paraneoplastic hypercoagulability, in oncological patients, an overall increase in circulating platelets has been reported. This thrombocytosis (defined as circulating thrombocytes of more than 450.000/mm^3^) is determined by aberrant paracrine signaling via a high circulating level of interleukin 6 (a by-product of the tumor microenvironment) and thrombopoietin (produced by the liver) [65].

Case control studies reported associations between thrombocytosis in primary care and certain underlying cancers (lung, renal, uterine, colorectal) [66,67,68,69]. In the case of other cancer types, however, this association was not found [70].

We have considered for our fuzzy logic system only two membership functions for *PLT*, one comprising the normal values interval and the other, which we considered high risk, the interval close to the upper limit of the normal values interval and all values above. This decision was taken because thrombocytopenia associated with solid tumors is highly unlikely in the absence of bone marrow infiltration, chemotherapy toxicity, or end-stage disease, all those particular cases being outside the scope of the present study.

- Alkaline phosphatase

Alkaline phosphatase (ALP) comprises a group of membrane-bound metalloenzymes that catalyze the hydrolysis of phosphate esters in an alkaline environment. It is widely distributed in the body, with the highest concentrations being found in the liver and bone marrow and in lesser amounts in the intestine, placenta, and leukocytes [71]. The metabolic role of ALP is yet to be uncovered. Data available suggest a role in lipid transportation in the intestines, bone transformation processes, and cell division through the cleaving of phosphate groups from nucleotides [72].

From a clinical standpoint, the hepatic and bone-produced isoenzyme (also referred to as nonspecific) is the most important, representing 80% of serum ALP. In certain situations, the intestinal isoenzyme can also determine the elevation of serum ALP, for example after the consumption of lipid-rich meals in patients with 0 and A blood type [73]. There are gender and age fluctuation in the normal serum range which have been described, as well as a direct correlation with smoking and body mass index, and an indirect correlation with height, although the reason for these variations remains uncertain [74].

With this in consideration, it becomes obvious why primary or secondary hepatic or bone tumors will determine high serum values of ALP. Through the observation of elevated ALP in cancer patients, in the presence or absence of metastasis, it has been hypothesized that ALP might be involved in carcinogenesis or progression [75].

In the specific case of colorectal patients, elevated serum ALP has been observed in the presence of hepatic metastases [76]. Patients with elevated serum ALP, in the absence of metastasis at the moment of diagnosis, are 5.5 times more likely to develop hepatic metastases, thus making this parameter a negative prognostic factor. Elevated values also reflect the progression of the neoplastic process, higher values being correlated with later stages [77].

We have considered for our fuzzy logic system only two membership functions for *ALP*, one comprising the normal values interval and the other, which we considered *high* (risk), the interval comprising the values above the upper limit of this interval. This decision was taken because hypophosphatasia is an extremely rare condition, and is not described as a paraneoplastic syndrome. The choice of membership function intervals, as is the case of the other parameters, is based on the existing medical data.

- Hemoglobin

Anemia is a known complication of cancer, although the exact prevalence is difficult to estimate. One of the most important reasons is the fact that studies usually focused on cancer patients with severe anemia (Hemoglobin level under 8 G/dL), which is thought to have the greatest physiological consequences. Newer clinical data suggest that a hemoglobin level below 12 g/dL can highly impact clinical prognosis and the quality of life [78].

There are numerous causes that contribute to anemia: hypersplenism, hemolysis, renal disfunction with consecutive reduced erythropoietin production, nutritional deficiency, systemic inflammation, bone marrow damage, and treatment related toxicity. When referring to gastrointestinal solid tumors, another important cause of anemia is represented by tumor bleeding, which usually goes unnoticed by the patient, making anemia the hallmark sign which determines referral [79]. In the case of colorectal cancer, age, tumor size, and tumor site are important factors influencing hemoglobin levels, whereas histological type and staging appear not to have an overall impact [80].

The European Cancer Anemia Survey (ECAS), a large prospective study that enrolled more than 15,000 patients, concluded that 39% of all patients with colorectal cancer presented anemia at the time of diagnosis [81].

In our fuzzy system, we have assigned three membership functions to assess the risk of PC occurrence in regard to Hemoglobin values. The high-risk membership function was designed to overlap with the normal values range, whereas the representation of the average risk membership function overlapped with the lower half of the normal values interval and anemia, with hemoglobin levels above 8 G/dL. Due to the lack of medical data regarding the association between anemia and isolated PC, this design choice has been taken in accordance both with the medical database of the study, as well as with indirect literature data. High grade adenocarcinomas are characterized by rapid tumor progression and metastasis, thus more likely to be diagnosed in late stages, with PC and/or organ metastasis and less likely to be accompanied by anemia due to occult tumor hemorrhage. The third membership function, low risk, was overlapped over the hemoglobin values characteristic of moderate and severe anemia. This might appear counterintuitive, but it is within the study scope range. Moderate and severe anemia, as an isolated modification of the routine blood tests, prompts by itself swift and complex diagnostic tests and treatment, therefore these patients will not require this particular multifactorial probabilistic diagnostic tool.

### The Fuzzy Logic Approach

Fuzzy logic is an approach that studies reasoning systems where the notions of truth and false are considered in a graded fashion. Fuzzy logic analyzes the vagueness in natural language and several other application domains, including the medicine field. Fuzzy logic is a soft computing technique that tolerates vagueness, consequently providing very good solutions [82].

Typically, process modeling and analysis systems can perform properly only when relationships between the variables can be determined. But the Fuzzy logic methods are an alternative design for complex processes and systems where the dynamics cannot be described by traditional mathematics equations due to their intrinsic difficulty and vagueness so therefore the ease of evaluation is one of the reasons of choosing it [83,84]. Linguistic variables used in the fuzzy approach do not describe numerical data, but, by the membership functions, are scaled between zero and one [84].

Operations performed with fuzzy variables and associated fuzzy rules are based not on precise models of the process, but on understanding physical phenomena, such as IF -THEN.

The systems based on fuzzy techniques are more flexible than conventional systems because the change in the deduction rules of order size can be done by adding new language variables [85].

The aim of a fuzzy inference system is to draw a conclusion based on the possibly uncertain information. Due to linguistic and numerical requirements, the fuzzy modeling process must make a compromise between the precision and interpretability so basically, the fuzzy approach is to provide high numerical precision while incurring as little as possible a loss of linguistic descriptive power [86].

A fuzzy model is built based on knowledge from a human expert, so fuzzy modeling supposes to identify the parameters of a fuzzy inference system in order to provide the desired behavior of the system. The task becomes more difficult once the available data is incomplete, so it is necessary to use automatic approaches to fuzzy modeling. Another major problem in fuzzy modeling is the size of the problem, which means that computational requirements increase exponentially with the number of variables [87].

Typically, there are two main types of fuzzy inference systems, Mamdani and Sugeno. The Mamdani type inference system represents a method widely accepted because it entails expert knowledge and allows to represent the expertise in a more intuitive, more human-like manner, and based on these considerations, the Mamdani—type is used within fuzzy modeling [88].

Fuzzy reasoning supposes two distinct parts: evaluating the rule antecedent (the IF part of the rule) and implication or applying the result to the consequent (the THEN part of the rule).

According to the classical rule-based systems, if the rule antecedent is true, then the consequent is also true, while for the fuzzy systems (where the antecedent is a fuzzy statement), all rules fire to some extent, meaning it fires partially. If the antecedent is true to some degree of membership, then the consequent is also true to the same degree. The value of the output or a truth membership grade of the rule consequent can be estimated directly from a corresponding truth membership grade in the antecedent [40].

A fuzzy rule can have multiple antecedents. All parts of the antecedent are computed simultaneously and solved in a single number, using a fuzzy set operation. The consequence of a fuzzy rule may include several parts. If so, all parts of the consequent are equally affected by the antecedent. In general, a fuzzy inference system incorporates not one, but several rules that describe the knowledge.

The output of each rule is a fuzzy set, but usually, it is necessary to obtain a single number representing the fuzzy system output. In other words, we want to get a precise solution, not a fuzzy one. To obtain a single crisp solution for the output variable, a fuzzy inference system first aggregates all output fuzzy set into a single output fuzzy set, and then defuzzifies the resulting fuzzy set into a single number.

This fuzzy model has been designed using empirical data gathered from patients presenting early PC. Each of the parameters chosen characterizes a different side of the complex interaction between cancer and the host organism. By doing so, this fuzzy model represents a multi-sided approach in the diagnosis of early PC. After the fuzzification of the initial data, a set of rules were designed, each rule assigned with a certain degree of truth. The membership functions, rules, and degrees of truth have been designed using medical experience, literature data, and patient data. If most of the membership functions and rules directly derive from the medical studies, the membership functions and rules of the parameter *age* have been adapted to our dataset and indirectly supported by epidemiology. At a first glance, from a statistical standpoint, the two sets appear identical. This however might be a misinterpretation. In the PC dataset, a total of 144 patients have been subjected to the inclusion and exclusion criteria, with only 73 patients comprising the final data set. The total number of cases with CRC was 1990, and after inclusion and exclusion criteria were applied, only a matching set of 73 patients were selected. This selection was done in a quasi-random, the matching referring solely to the localization of the primary tumor. The reason behind this choice is the fact that right-sided colon and rectal cancer is associated with PC more often than other localization [15]. By randomizing and choosing the witness dataset solely by localization of the primary tumor, inaccuracies regarding age interval can be expected. Considering this, a second reason for choosing the high-risk interval for younger patients was the histological type. The younger the patient, the more likely the occurrence of a more aggressive, invasive histological type, and late-stage diagnosis [43,44]. This particularity regarding CRC in young patients can also explain the trend in Hb levels in our data sets: through a faster cancer progression, the rate and volume of tumoral hemorrhage is reduced, thus leading to higher Hb values. This possible explanation reiterates the complexity and interdependence of various systems in the cancer-host interaction.

The wide range of carcinomatosis probability reflects the basic non-Boolean approach which characterizes fuzzy logic. Our proposed fuzzy model only generates a probability value, the higher the values imposing a quicker referral to more specialized centers for a complex multidisciplinary treatment approach. Certain parameters from the original datasets were left out because they did not meet the scope of this study. For example, histological type requires a biopsy which already means the patient is high up on the diagnosis-cancer care pathway. Same with localization of the primary tumor, staging, or specific blood markers. The aim was to develop a fuzzy model which could be used even in primary care with minimal or routine blood tests.

There are certain limitations of the current study. The most important ones refer to the limited number of cases due to rigorous inclusion criteria. Larger patient databases could help perfect the membership functions and rules. Another possible bias is part of the scope of the study itself. By using basic blood tests, we increase the availability and feasibility of this approach. However, all the parameters chosen are highly nonspecific. Alteration of their values can occur in numerous other pathologies, therefore, initial data generated should be carefully interpreted from a clinical standpoint. In order to compensate for this possible bias through a multisided approach, we have chosen five parameters, each reflecting a different system affected in the presence of cancer. In the presence of CRC suspicion, the alteration of the homeostasis of all systems reduces as much as possible false positive results occurrence.

## 5. Conclusions

Peritoneal carcinomatosis can occur in the progression of every colorectal malignancy. It is still associated with poor prognosis and low quality of life. Patients with low burden PC can be eligible for complex multidisciplinary treatment options or benefit more from conventional chemotherapy alone. However, detection of early PC is extremely difficult without invasive diagnostic tools, such as exploratory laparoscopy. The present study uses routine blood tests and a fuzzy logic approach to positively identify a high probability of PC in patients with CRC. The novelty of the proposed approach resides in the attempt to identify systemic differences between multifocal and single-site cancer progression. Also, this is a first-time approach to using soft computing in the diagnosis of PC using blood tests. Also, this method can be improved and integrated with medical informatics software, decreasing referral time, and decreasing morbidity and mortality.

The fuzzy model represents an attempt to provide a quite comprehensive description and assessment of the probability of carcinomatosis occurrence based on five dimensions. The work submits attention to the link between the *age*, *Hb*, *CRP*, *PLT*, and *ALP*, and the specific quantitative alteration of these parameters in the presence of peritoneal carcinomatosis, which is an innovative approach as regards the literature in the field. Using linguistic variables and linguistic rules, the model provides quantitative measures of carcinomatosis diagnostic.

This represents a novel approach to the issue of early noninvasive diagnostic of peritoneal carcinomatosis of colorectal origin. Although still improvable and in need of tuning with large data sets, it proves the feasibility of a future valuable diagnostic tool.

## Figures and Tables

**Table 1:** Data analysis of patients with CRC and PC.

	Min	Max	Median	Std Deviation
Age (years old)	39	85	63	11.29
Hb (g/dL)	6.5	16.5	12.1	2.5
PLT (/mm^3^)	230,000	705,000	384,000	95,613.62
PCR (mg/dL)	0.48	26.63	6.2	6.685
ALP (U/L)	54	1103	95	190.81

Source: own processing based on data.

**Table 2:** Data analysis of patients with CRC.

	Min	Max	Median	Std Deviation
Age (years old)	29	91	69	12.92
Hb (g/dL)	5	15.8	10.3	2.61
PLT (/mm^3^)	155,000	561,000	301,000	129,710.3
PCR (mg/dL)	0.1	14.78	3.87	5.22
ALP (U/L)	43	384	80	56.83

Source: own processing based on data.

**Table 3:** *p*-value for matching parameter sets.

Parameter	*p*-Value
Age	0.011813529
Hb	0.069862
PLT	3.05357 × 10^−5^
PCR	0.001210458
ALP	2.46786 × 10^−9^

## Data Availability

The simulation files/data used to support the findings of this study are available from the corresponding author upon request.
